# Supplementary material for: A Global Screen for Assembly State Changes of the Mitotic Proteome by SEC-SWATH-MS
Source: Cell Syst. 2020 Feb 26;10(2):133–155.e6. doi: 10.1016/j.cels.2020.01.001 (PMC7042714; doi:10.1016/j.cels.2020.01.001)

Q9BZH6 | WDR11_HUMAN | WDR11 BRWD2 KIAA1351 WDR15
Monomer MW [kDa]: 136.685 Monomer expected elution fraction: 37

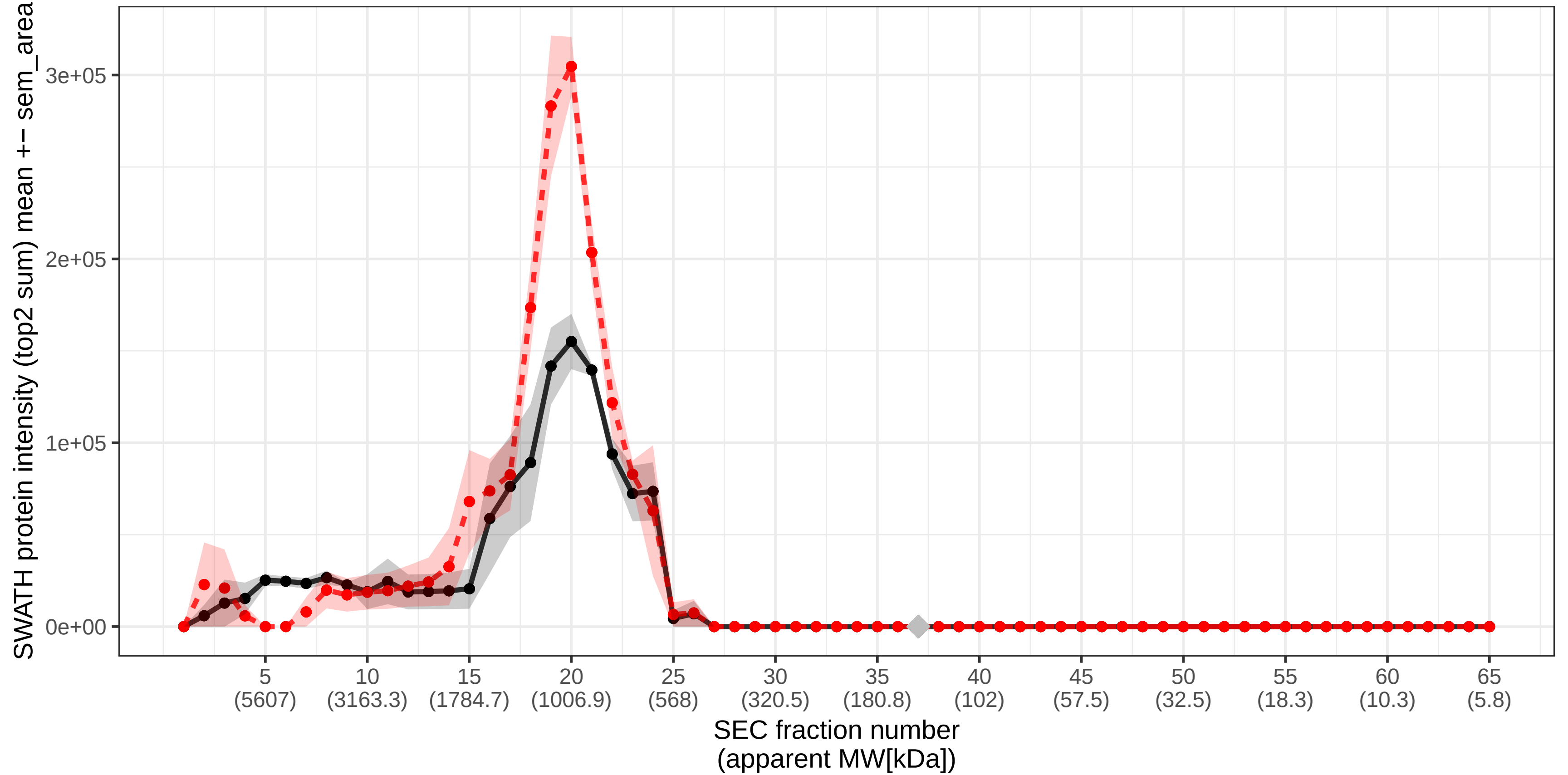

Supplement: Data S1. SEC-SWATH-MS Protein Chromatograms, Related to Figure 1 [file mmc6.zip › SECchrom_Q9BZH6_WDR11_HUMAN_WDR11_BRWD2_KIAA1351_WDR15.pdf]
